# Metabolomics Highlights Different Life History Strategies of White and Brown Rot Wood-Degrading Fungi

**DOI:** 10.1128/msphere.00545-22

**Published:** 2022-12-05

**Authors:** J. D. Castaño, N. Muñoz-Muñoz, Y. M. Kim, J. Liu, L. Yang, J. S. Schilling

**Affiliations:** a Bioproducts and Biosystems Engineering, University of Minnesota, Saint Paul, Minnesota, USA; b Marine and Coastal Research Institute, INVEMAR, Santa Marta, Colombia; c Pacific Northwest National Laboratory, Richland, Washington, USA; d Brookhaven National Laboratory, Upton, New York, USA; e Department of Plant and Microbial Biology, University of Minnesota, Saint Paul, Minnesota, USA; University of Wisconsin-Madison

**Keywords:** secondary metabolites, solid state culture, antioxidant activity, brown rot fungi, white rot fungi, wood decay

## Abstract

White and brown rot fungi efficiently deconstruct lignocellulose in wood, Earth’s largest pool of aboveground biotic carbon and an important natural resource. Despite its vital importance, little is known about the metabolomic signatures among fungal species and nutritional modes (rot types). In this study, we used GC-MS metabolomics in solid wood substrates (*in planta*) to compare brown rot fungi (Rhodonia placenta and Gloeophylum trabeum) and white rot fungi (Trametes versicolor and Pleurotus ostreatus) at two decay stages (earlier and later), finding identifiable patterns for brown rot fungi at later decay stages. These patterns occurred in highly reducing environments that were not observed in white rot fungi. Metabolomes measured among the two white rot fungi were notably different, but we found a potential biomarker compound, galactitol, that was characteristic to white rot taxa. In addition, we found that white rot fungi were more efficient at catabolizing phenolic compounds that were originally present in wood. Collectively, white rot fungi were characterized by measured sugar release relative to higher carbohydrate solubilization by brown rot fungi, a distinction in soluble sugar availability that might shape success in the face of “cheater” competitors. This need to protect excess free sugars may explain the differentially high brown rot fungal production of pyranones and furanones, likely linked to an expansion of polyketide synthase genes.

**IMPORTANCE** Despite the ecological and economic importance of wood-degrading fungi, little is known about the array of metabolites that fungi produce during wood decomposition. This study provides an in-depth insight into the wood decomposition process by analyzing and comparing the changes of >100 compounds produced by fungi with metabolic distinct nutritional modes (white and brown rot fungi) at different decay stages. We found a unique pattern of metabolites that correlated well with brown rot (carbohydrate selective mode) in later decay. These compounds were in line with some of the physiochemical and genetic features previously seen in these fungi such as a faster sugar release, lower pH, and the expansion of polyketide-synthase genes compared to white rot fungi (lignin-degrading mode). This study provides spatiotemporally resolved mechanism insights as well as critical groundwork that will be valuable for studies in basic biology and ecology, as well as applied biomass deconstruction and bioremediation.

## INTRODUCTION

Wood-degrading fungi play an essential role in global nutrient cycles as they release large amounts of plant-sequestered carbon (present in the lignocellulose complex) and make available the nutrients bound in plant tissues (1). Due to their special abilities to decompose the recalcitrant materials present in wood, they have also been used for different applications such as bioremediation, biofuel production, and the pulping industry (2–5), There are different types of wood-degrading organisms with variable abilities to decompose wood, with brown rot and white rot fungi being most common, at least in temperate environments. Brown rot fungi typically degrade wood faster than white rot fungi, rapidly consuming the cellulose and hemicellulose components and primarily modifying and oxidizing lignin without significant removal (6–9). The mechanisms brown rot fungi use to achieve this leave distinct signatures that are outwardly observable; wood rapidly loses its strength and becomes brown, crumbly, and fissured across the grain (cubical checking) (10). On the contrary, white rot fungi degrade lignin extensively during wood decay, which they carry out simultaneously with cellulose or selectively, delaying cellulose degradation. This distinct white rot approach leaves moist, spongy, and stringy wood residues that are white rather than brown (10).

In addition to these signatures of rot type that are observable in decomposing wood, a variety of genetic factors distinguish brown and white rot fungi. White rot fungi rely heavily on carbohydrate-degrading enzymes (CAZy) and high-redox lignin oxidoreductases (11). Brown rot fungi, which evolved multiple times from white rot ancestral clades (convergent evolution), shed lignin-degrading genes and lost an average of >60% of CAZys (1, 12, 13), with an apparent upgrade in efficiency when comparing decay rates in pure culture (9). The use of low molecular weight oxidants (i.e., Fenton-derived hydroxyl radicals) and an overexpression of the few CAZys retained in brown rot fungi compensate this loss (13).

Both factors, (i) fungal metabolites derived from distinct genomes, and (ii) wood-derived compounds that vary depending on rot type, should leave “signature” metabolite patterns throughout decay. One would expect that the compounds secreted by white rot fungi (e.g., organic acids and secondary metabolites) and the molecules released from white-rotted wood (e.g., solubilized sugars and polyphenolics) would differ from patterns during brown rot. In addition to predictable differences in oxalic acid, soluble sugars, and lignin-derived compounds (10), mapping metabolomic signatures over the course of wood decay promises to direct attention to other less-clear aspects of these decay mechanisms. One example is the significant, but poorly understood expansion in reducing polyketide synthases in brown rot fungi, which could code additional pyranones, furanones, quinones and other polyketide-related secondary metabolites that have not yet been assigned a function (14). These compounds may be correlated with antioxidant activity specific to the brown rot mechanism and meant to quench reactive oxygen species (ROS) at later decay stages (15). Extensive metabolite patterns in decaying wood, however, have rarely been reported using global “omics” approaches, and have been focused, in those few cases, only on white rot species (16–19).

In this study, we focused a metabolomics effort on this fungus-wood interplay using brown and white rot fungi from distinct evolutionary clades. We grew these fungi directionally across aspen wood wafers, and then we sectioned and analyzed the wafers in a way to separate earlier (early) from later (late) decay stages, as well as separate compounds by type (e.g., polarity and volatility). Results showed that brown rot fungi presented an identifiable metabolite signature with a highly reducing environment at later decay stages and revealed some potential biomarkers unique to each type of decay. For instance, some furanones and pyranones were highly abundant in brown rot fungi, probably as result of the polyketide synthase gene expansion in these fungi. These metabolite profile data not only highlight distinctions between rot types, but also show real-time interplay between secreted compounds and substrate solubilization. This will help us, and others, build better genome regulation models and better understand how these fungi react to and control their own progress when decaying woody substrates.

## RESULTS AND DISCUSSION

### Distinct metabolite signatures for brown and white rot fungi.

**FIG 1:**
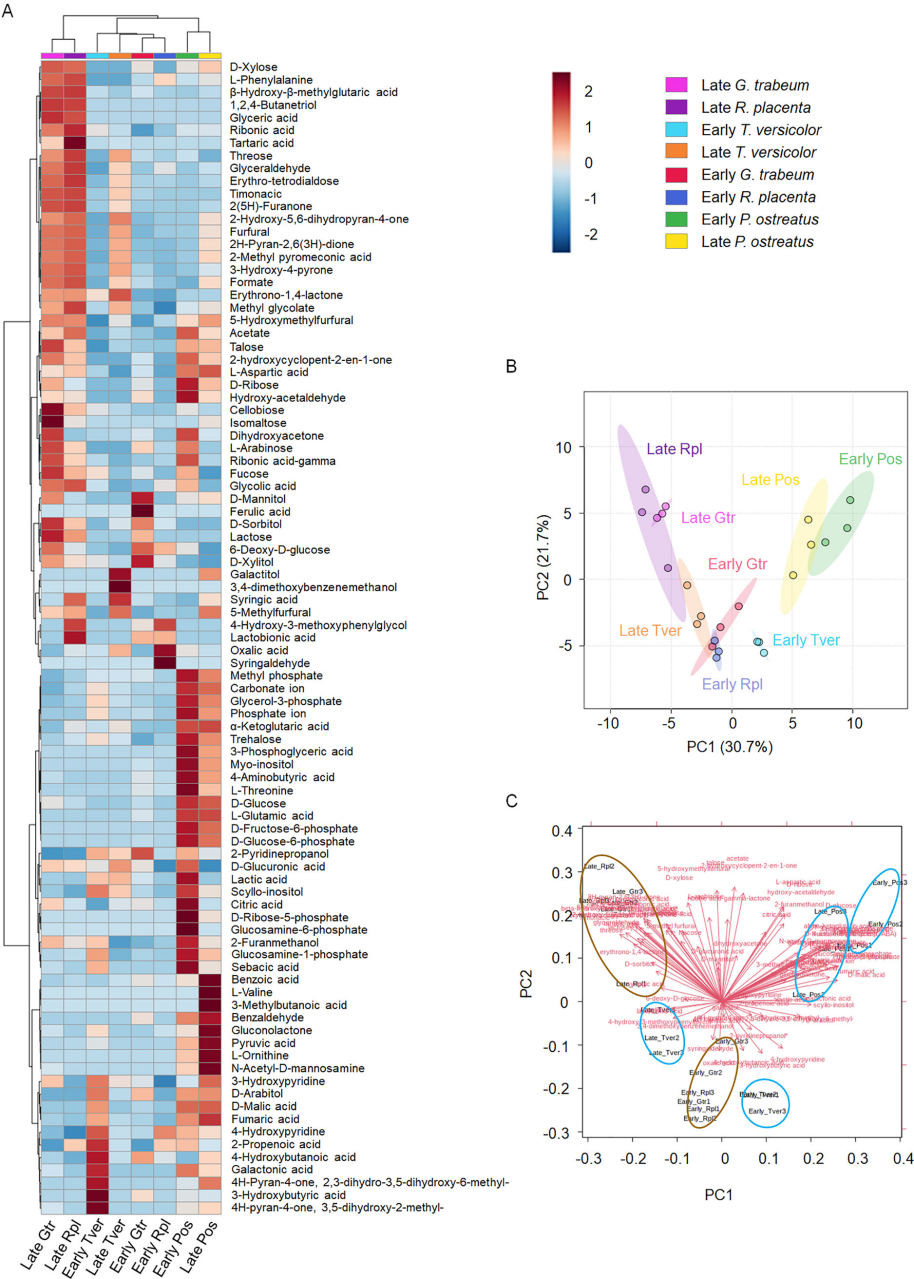
A. Heatmap showing 95 metabolites and their distribution among wood-decaying fungi (*G. trabeum*, *R. placenta*, *P. ostreatus*, and *T. versicolor*) at different stages of decay (early and late). Wood metabolite intensities were subtracted from the samples and subsequently normalized by the ergosterol content in each section, as described in the methods. B. Principal component analysis (PCA) from different metabolites quantified in different fungi at different stages of decay C. PCA biplot. The arrows suggest that some metabolites seem to be more common in early and late brown rot fungi. Early and late decay stages in *T. versicolor* also show some distinctive features. Finally, early and late decay stages in *P. ostreatus* cluster far away from the other fungi and share some common characteristics.

Extracellular metabolite studies of wood decay fungi are scarce, and some only compare white rot fungi (19). To address this issue, we conducted the first extracellular metabolomics study *in planta* to the best of our knowledge to try to understand some of the functional differences between brown and white rot fungi, measuring compounds secreted by fungi and solubilized from wood. First, through a PERMANOVA analysis (Table 1), we found that there were significant differences (*P* < 0.01) among the secreted metabolomes studied, with fungal species being the main factor driving these differences (40.3%), followed by decay stage (19.8%), and finally the interaction between these two factors (17.0%). Second, as the heatmap and principal component analysis (PCA) plots show (Fig. 1), the samples presented distinct metabolite profiles, which clustered depending on the fungal species and stage (early versus late) of decay. Remarkably, early and late Gloeophylum trabeum and Rhodonia placenta samples showed a higher level of similarity and clustered together as opposed to the early and late samples in white rot fungi. Indeed, the samples of the fungus Pleurotus ostreatus were distinctly different from all the other fungi and clustered together rather far away from the samples of the other white rot fungus, Trametes versicolor. Interestingly, the late decay samples of *T. versicolor* showed a higher similarity with the samples from late decay of *R. placenta* (as deduced from the PCA plot), which may reflect to their evolutionary relationship in the same order (*Polyporales*).

**TABLE 1:** PERMANOVA analysis of metabolite abundances across different fungal species and wood decay stages

Parameter	DF	Sum of squares	Mean squares	F model	R^2^	*P* value
Decay section (early or late)	1	438.1	438.1	13.86	0.20	1e−6
Fungus	3	889.2	296.4	9.38	0.40	1e−6
Decay section × fungus	3	375.0	125.0	3.95	0.17	4e−6
Residuals	16	505.6	31.6		0.23	
						
Totals	23	2208.0			1.00	

**FIG 2:**
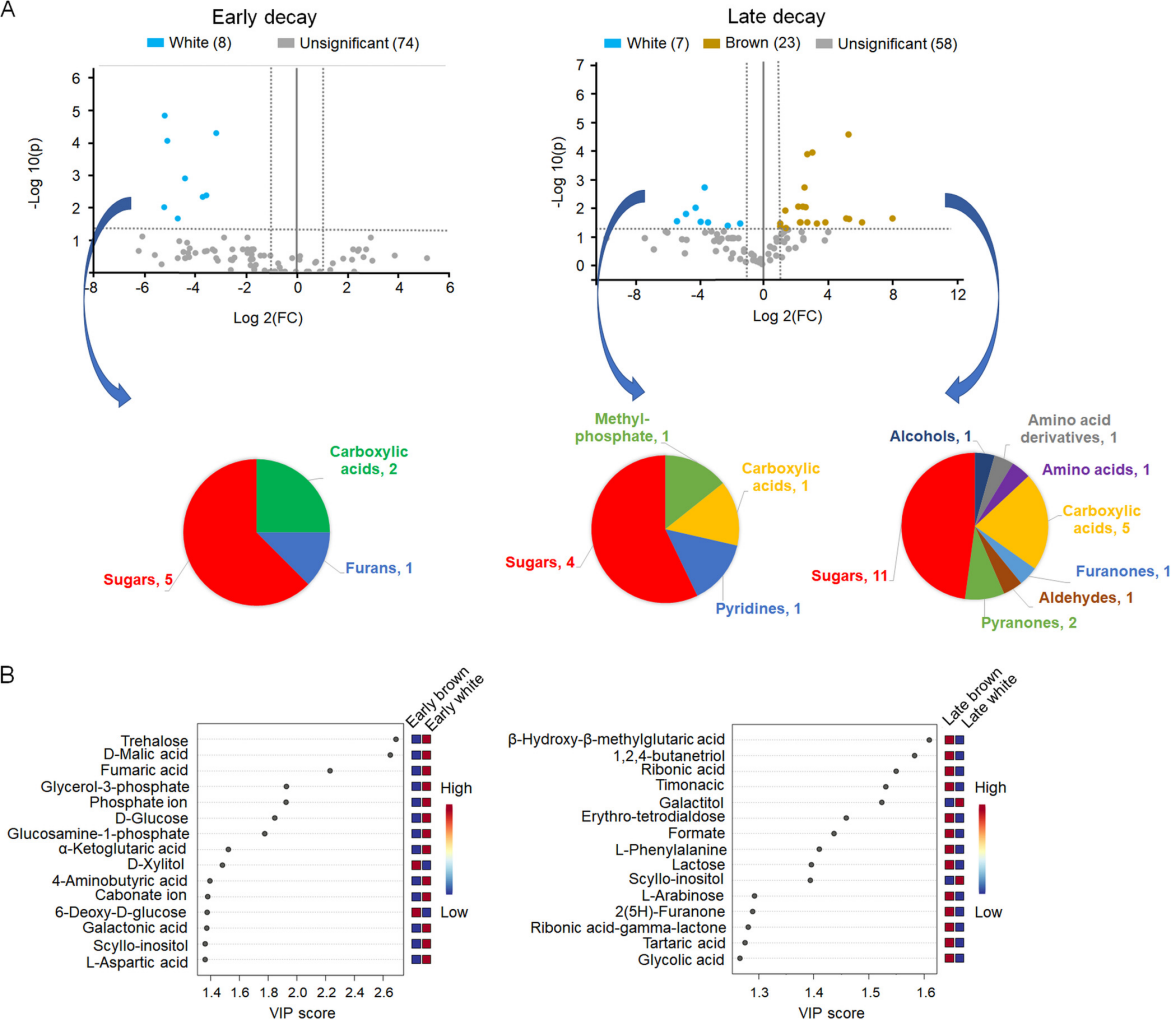
A. Volcano plots showing differentially abundant metabolites (FC ≥ 2, FDR <0.05) at early and late stages of decay in white and brown rot fungi. The types of compounds that were differentially abundant depending on the type of fungus and decay state are shown in the pie charts below the volcano plots. At early decay stages there were no compounds differentially abundant in brown rot fungi. B. Variable importance projection (VIP) scores show the main variables (compounds) associated with the type of decay studied (white or brown rot decay) at different decay stages (early or late). Only the top 15 compounds are shown.

Despite the differences between *T. versicolor* and *P. ostreatus* metabolomes, we found that there were some commonalities to white rot when comparing these samples with brown rot decay samples. The volcano plots in Fig. 2A show how in both early and late decay stages it was possible to find compounds that were significantly different between white and brown rot. In early white rot, there were 8 compounds at significantly higher abundance relative to early brown rot, but interestingly, there were no compounds at significantly higher abundance in early brown rot. In contrast, at late decay stages, we found 23 compounds that were significantly more abundant in brown rot compared to white rot, as opposed to only 7 more abundant in late-stage white rot compared to brown rot. This is in line with the higher similarity between the metabolite profiles shown in Fig. 1A for late decay stages in brown rot fungi as opposed to the stark difference between both early and late decay in *T. versicolor* and *P. ostreatus* metabolomes.

Sugars were the main compounds explaining these pattern distinctions, which is understandable given the distinct mechanisms for accessing and depolymerizing carbohydrates among these fungi. In early white rot decay, we observed 5 sugars (trehalose, scyllo-inositol, glucosamine-1-phosphate, galactonic acid, and glycerol-3-phosphate) that were differentially more abundant than in brown rot fungi. Glucosamine-1-phosphate is a sugar involved in the biosynthesis of the fungal cell wall, and considering that the two white rot fungi tested had a higher growth rate than the brown rotters (see Table S1 in the supplemental material), it makes sense that we found higher concentrations of this compound at early decay stages. On the other hand, galactonic acid (product of galactose oxidation), and scyllo-inositol are easily solubilized sugars that are typically released during early wood decay before considerable degradation of lignocellulose occurs (20).With respect to trehalose, this compound is generally produced by fungi early on to handle stress, being useful to respond to oxidative stress, osmotic stress, or heat shock (21); it probably starts accumulating first in white rot fungi due to their higher growth rate.

10.1128/msphere.00545-22.4 TABLE S1 Growth rates for different wood decaying fungi growing on aspen wood wafers. Download Table S1, DOCX file, 0.01 MB. Copyright © 2022 Castaño et al. 2022 Castaño et al. https://creativecommons.org/licenses/by/4.0/ This content is distributed under the terms of the Creative Commons Attribution 4.0 International license.

Interestingly, growth rate only correlates with higher wood decay rates within the same decay group. For instance, it has been shown that fast-growing white rot fungi cause faster wood decay compared to slow growers (22). However, brown rot fungi, which grow slower than white rot fungi, have consistently exhibited faster wood decay than white rotters, as evidenced by a higher weight loss rate, strength loss, carbohydrate degradation, and dilute alkaline solubility (DAS) relative to density loss (23, 24). In this study, this feature was also confirmed by the higher loss of cellulose crystallinity in brown rot (Fig. S2). The decrease of cellulose crystallinity implies the loss of a rigid hydrogen bond network, which makes amorphous cellulose more reactive and easier to degrade (25); thus, this important difference likely played a role in differences seen at later decay stages, as discussed later.

10.1128/msphere.00545-22.3 FIG S2 Representative X-ray scattering intensity I(q) plot. The intensity profiles are normalized to I(q = 0.02 Å-1) for I(q) and to the peak intensity for I(ɸ-ɸ0), where ɸ0 is the peak angular position. Blue lines correspond to undecayed samples while orange lines correspond to decayed samples. The measurements are the average of at least 5 points collected from three biological replicates. The crystallinity index (CI) is defined as the intensity ratio between the (200) peak and the amorphous background (Fig. S2 additional discussion). The intensity profiles are normalized to I(q = 0.02 Å-1) for I(q) and to the peak intensity for I(ɸ-ɸ0), where ɸ0 is the peak angular position. The motivation behind the I(q) normalization is that the overall architecture of the cell walls, and therefore the scattering intensity at low q, is not expected to change significantly during fungal digestion. On the other hand, the peak at q~0.2 Å-1 that corresponds to the spatial correlation between cellulose fibrils is expected to diminish as the number density of the fibrils is reduced. The high-q portion of the I(q) profile corresponds to the well-known crystalline cellulose structure, superposed atop the scattering from amorphous structural components. The CI provides a qualitative indicator of the amount of crystalline cellulose present in the sample. Any changes in the amorphous background results in a shift of overall scattering intensity but does not alter the CI. The angular intensity profile is indicative of the orientation distribution of cellulose fibrils. However, a change in the angular distribution is not necessarily a consequence of fungal digestion since the fibril orientation in intact cell walls may not necessarily be the same. Download FIG S2, TIF file, 1.4 MB. Copyright © 2022 Castaño et al. 2022 Castaño et al. https://creativecommons.org/licenses/by/4.0/ This content is distributed under the terms of the Creative Commons Attribution 4.0 International license.

Aside from sugars, two organic acids (fumaric acid and d-malic acid) and 2-furanmethanol were also found to be differentially more abundant in white rot than in brown rot at early decay stages. Organic acids have been considered important for competitive advantage for several wood-degrading fungi because they lower the pH, modifying it to more optimal values for their enzymes (26); additionally, they also contribute to enhanced weathering and metal detoxification in natural forest environments (27). Likewise, 2-furanmethanol also offers competitive advantages to fungi by inhibiting the growth of other organisms (28) and acting as an antioxidant (29). However, in view of the low antioxidant activity registered (as will be shown later), this role is probably not particularly important for this molecule in white rot fungi.

At late decay stages, there were also several sugar compounds that were more abundant in white rot fungi (glycerol-3-phosphate, galactitiol, scyllo-inositol, and d-arabitol), followed by other compounds such as α-keto-glutaric acid, methyl phosphate, and 4-hydroxypyridine. d-arabitol, as well as trehalose (more abundant in early white rot decay), have been previously identified as potential biomarkers for fungal presence/absence in a variety of conditions, including wood decay (30). Our results suggest that these compounds might reflect white rot fungal presence/absence, specifically because they are significantly more abundant in this type of decay. Moreover, galactitol was found only in white rot fungi. Although these assertions would need a broader survey of white rot fungi, our findings suggest galactitol could be a useful biomarker for white rot type of fungal decay.

In brown rot fungi, we also found several sugar compounds as significantly more abundant at late decay stages, which is in line with the higher loss of cellulose crystallinity observed in the samples (Fig. S2) that makes cellulose more reactive. Additionally, other compounds such as carboxylic acids and pyranones were also more abundant (Table S2). Also, 2 out of 11 sugars were detected only in brown rot fungi (lactose and glyceric acid). Glyceric acid has been reported before in brown rot-treated and Fenton-depolymerized cellulose as a compound released due to the oxidative cleavage of glucosyl residues (26). Other sugars such as l-arabinose, fucose, and d-xylose are the result of hemicellulose degradation (31). Likewise, d-xylitol is likely produced in the metabolism of xylose after the reduction of the latter by a xylose reductase (32). Two pyranones (2H-pyran-2,6[3H]-dione and 3-hydroxy-4-pyrones) and one furanone (2[5H]-Furanone) were also significantly more abundant, which is likely related to the expansion of polyketide synthase genes in brown rot fungi with respect to white rot fungi (14). These compounds are secondary metabolites that typically confer some competitive advantages against other organisms. Several pyranone and furanone derivatives have shown antibacterial and antifungal activity in different studies (33, 34). We also observed a higher abundance of erythro-tetrodialdose at late decay stages in brown rot fungi. Similar compounds such as threonate and erytronate have been identified in fungi under oxidative stressing conditions (16). Although their origin or function is not clear, some reports suggest they might be produced by the reaction between H_2_O_2_ and ascorbate (35), in which case a similar function could be hypothesized for erythro-tetrodialdose. Finally, there is also a higher of number of carboxylic acids significantly more abundant in late brown rot decay (5) compared to white rot fungi (1). This is consistent with observations that brown rot fungi create and maintain lower pH environments than white rot fungi (36–38).

10.1128/msphere.00545-22.5 TABLE S2 Compounds that were significantly (*P* < 0.05) more abundant (FC ≥2) in either early or late decay stages in brown rot decay (*G. trabeum* and *R. placenta*) and white rot decay (*P. ostreatus* and *T. versicolor*). Download Table S2, DOCX file, 0.01 MB. Copyright © 2022 Castaño et al. 2022 Castaño et al. https://creativecommons.org/licenses/by/4.0/ This content is distributed under the terms of the Creative Commons Attribution 4.0 International license.

To identify the most important compounds associated with a specific type of decay, we used the variable projection scores (VIP) obtained from a partial least squares-discriminant analysis (PLS-DA) model (Fig. 2B), where values higher than 1.0 typically show meaningful correlations between the metabolites and the characteristics under study (39). For early decay, we found two compounds that were correlated with brown rot fungi namely, d-xylitol and 6-deoxy-d-glucose. In white rot fungi, we found several compounds with high correlation scores, such as trehalose, d-malic acid, or fumaric acid. Several of these compounds were also present in the significantly more abundant compound list discussed before (Table S2). In contrast, at late decay stages, most of the compounds with the highest VIP scores correlated with brown rot fungi, with compounds such as β-hydroxy-β-methylglutaric acid, 1,2,4-butanetriol, ribonic acid, or timonacic at the top of the list. Nonetheless, the sugar alcohols galactitol and scyllo-inositol showed a high correlation score with white rot fungi.

The general trend observed between brown and white rot fungi suggests that white rot fungi release large amounts of sugars from the substrate early but then control release to better match their own nutritional needs. This could be the product of a higher growth rate and a reflection that they are prone in nature to microbial competition, where excessive sugar release risks “theft.” Brown rot fungi, on the other hand, may face less “cheater” competition as conifers associate more common in temperate and boreal forests (1). This may explain why brown rot fungi are able to use ROS to indiscriminately depolymerize wood and then secrete larger amounts of a less diverse set of glycosyl hydrolases (13). This results, at later decay stages, in the release of large amounts of sugars. If taken as functional traits, these sugar release strategies may play a major role in niche differentiation between fungi with these dramatically different approaches (white versus brown rot) to decomposition.

### Different metabolite signatures in early and late decay stages during wood decay.

**FIG 3:**
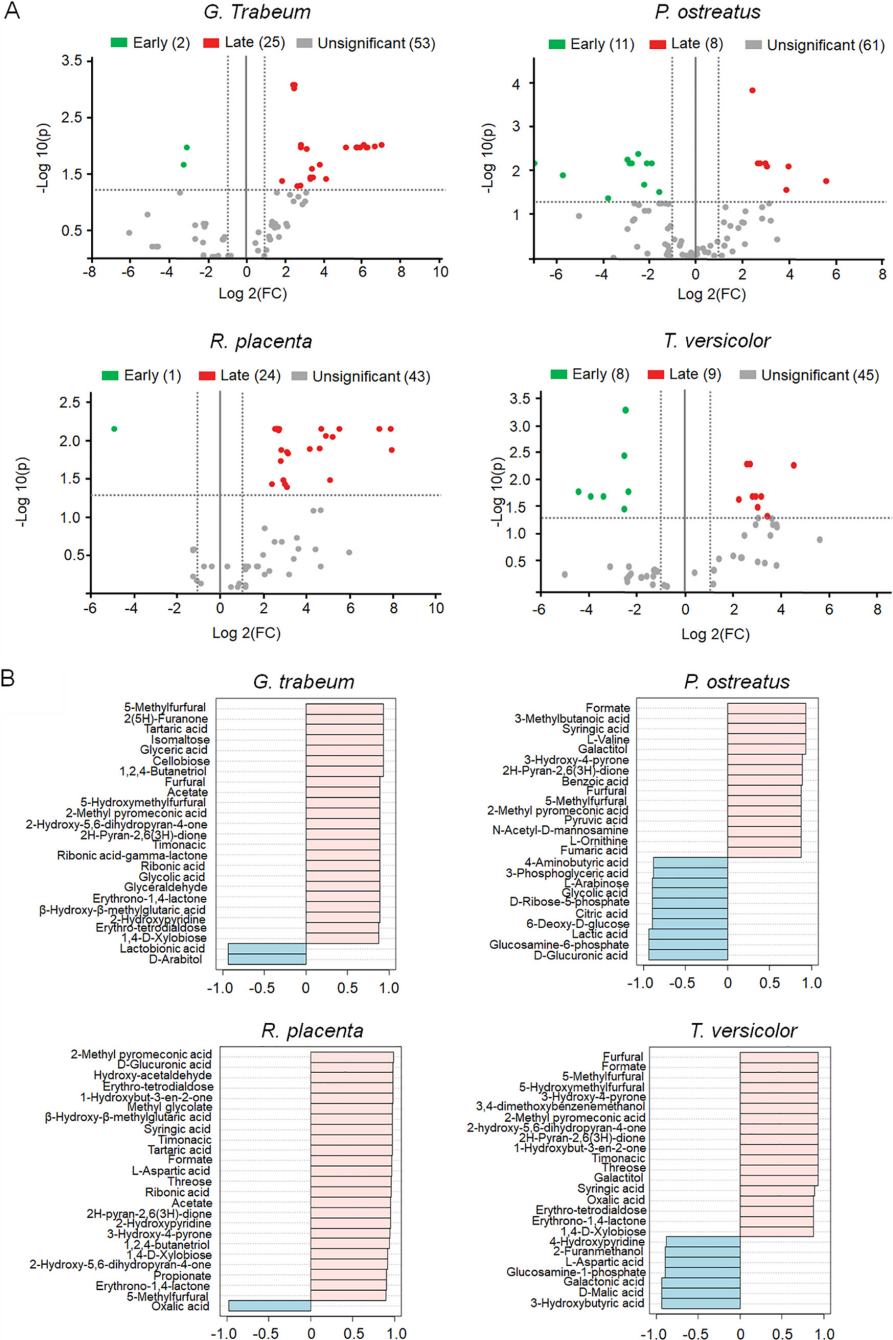
A. Volcano plots showing differentially abundant compounds (FC > 2.0, FDR < 0.05) at early and late decay stages in four different wood-decaying fungi, namely, *G. trabeum*, *P. ostreatus*, *R. placenta*, and *T. versicolor*. B. Spearman correlation coefficients for *G. trabeum*, *P. ostreatus*, *R. placenta*, and *T. versicolor*. Negative numbers close to −1.0 indicate a strong correlation with early decay stages. Conversely, positive numbers close to 1.0 indicate a strong correlation with late decay stages.

Early and late decay stages in each fungus also showed very distinctive metabolite profiles (Fig. 3A) with many compounds being differentially abundant in each stage (Fig. 3A). In *G. trabeum*, we found two significantly more abundant compounds at early decay, d-arabitol and lactobionic acid, that also showed a high correlation (close to 1.0) with early decay according to the Spearman correlation (Fig. 3B). Lactobionic acid could be important to lower the pH at early decay in *G. trabeum* as a complement or alternative to oxalic acid, the most abundant acid found at early decay in *R. placenta*. At late decay stages, there were several sugar compounds (8) significantly more abundant in *G. trabeum*, followed by carboxylic acids (4), pyranones (4), furans (3), and furanones (2) as the most representative compounds (Table S3). Several of these compounds are the result of the hydrolysis of the lignocellulosic complex. For instance, the increase in acetate is due to the hydrolysis of acetylxylan in hemicellulose (40). Likewise, further degradation of hemicellulose releases xylose and other 5-carbon sugars that can end up forming furans such as furfural, 5-methylfurfural, and 5-hydroxy-methylfurfural (40). Different types of furans were found to be significantly more abundant at late decay stages in all fungi except *T. versicolor*. Previous reports have also shown an increase of furan compounds at late decay stages; for instance, the fungi *Trichaptum abietinum* and *Phlebia radiata* produced methyl 3-furoate in highly degraded samples of spruce (41). The accumulation of furfurals may play a role in slowing down fungal growth (42), in turn prompting the colonization of new substrates and perhaps inhibiting competition. In line with this, we observed 2-furanmethanol accumulating faster in white rot fungi at early decay stages, which agrees with their faster growth.

10.1128/msphere.00545-22.6 TABLE S3 Compounds that were significantly (*P* < 0.05) more abundant (FC ≥2) in either early or late decay stages in *G. trabeum*, *R. placenta*, *P. ostreatus*, and *T. versicolor*. Download Table S3, DOCX file, 0.02 MB. Copyright © 2022 Castaño et al. 2022 Castaño et al. https://creativecommons.org/licenses/by/4.0/ This content is distributed under the terms of the Creative Commons Attribution 4.0 International license.

In *R. placenta*, we only found one compound to be significantly more abundant at early decay stages, oxalic acid, which is crucial at this stage for modulation of ROS and for Fe^3+^ chelation, which helps mobilize iron (43). Higher concentrations of oxalic acid facilitate a more effective control of iron reduction (44), limiting the potential negative effect of an excess of ROS, which is particularly important during the early stages of decay as more ROS-producing genes are upregulated at this point (45). At late decay stages, several sugars (5), carboxylic acids (5), pyranones (4), and other compounds (Table S3) showed to be more significantly abundant than in early decay. An increase in formate was observed in *R. placenta* as well as *P. ostreatus*, and *T. versicolor* at late decay, which is probably the result of oxalate decarboxylase transforming oxalate to formate and CO_2_ to detoxify the growth media from the negative effects of an excess of oxalate (46). Syringic acid, a phenolic compound, was also significantly more abundant at late decay in all fungi except *G. trabeum*. This result suggests that *G. trabeum* is probably able to either catabolize or modify this compound faster than the other fungi so that it does not accumulate in the growing environment, which implies an advantage for *G. trabeum*, considering that phenolic compounds typically inhibit fungal growth (47). Other compounds that were only significantly more abundant in *R. placenta*, *P. ostreatus*, and *T. versicolor* but not in *G. trabeum* included timonacic, erythrone-lactone, and 2-methyl pyromeconic. Presumably, these compounds could be conferring some competitive advantage to these fungi against other organisms. From a biotechnological utilization standpoint, pyromeconic acids and their derivatives are known to possess bioactivity such as inhibition of influenza endonucleases (48).

Contrary to brown rot fungi, *P. ostreatus* and *T. versicolor* exhibited a higher number of compounds as significantly more abundant in early wood decay. In the case of *P. ostreatus* there were 8 sugars and 3 carboxylic acids at higher concentrations at early decay, while *T. versicolor* showed a more assorted group of compounds such as sugars (2), carboxylic acids (3), furans (1), alcohols (1), and pyridines (1) (Table S3). Most of the sugars found in both cases are related to the early degradation of hemicellulose, suggesting (as mentioned before) that white rot fungi are be able to release sugars from hemicellulose earlier than brown rot fungi during wood decay. Another compound significantly more abundant in *P. ostreatus* was 4-aminobutyric acid. Although there are no reported functions for this compound during wood decay, this molecule has been related to several health benefits before, highlighting one of the many biotechnological potentials of this fungus (49).

Finally, at late decay stages there was a rather low number of compounds significantly more abundant in *P. ostreatus* (8) and *T. versicolor* (9) than in early decay, particularly compared to brown rot fungi where more than 20 compounds were significantly more abundant at late decay (Table S3). Three compounds were common to both white rot fungi, syringic acid, formate, and galactitol, with the latter being present only in these two fungi and not in the brown rot. Lastly, we found that veratryl alcohol (3,4-dimethoxy-benzenemethanol) was only found in the late decay caused by *T. versicolor*, where it was significantly more abundant compared to early decay. This compound has been associated with a variety of roles such as the mediation of the oxidation of some lignin substrates, and the protection of lignin peroxidase from degradation by H_2_O_2_ (50, 51). We recently reported that *T. versicolor* expresses a higher number of class II peroxidases (18) compared to *P. ostreatus* (4) in a similar experimental setup (13). This higher number of peroxidases might justify the presence of additional protective compounds such as veratryl alcohol to carry out decay by *T. versicolor*.

### Metabolites in undecayed sections that are rapidly consumed as fungal decay progresses.

**FIG 4:**
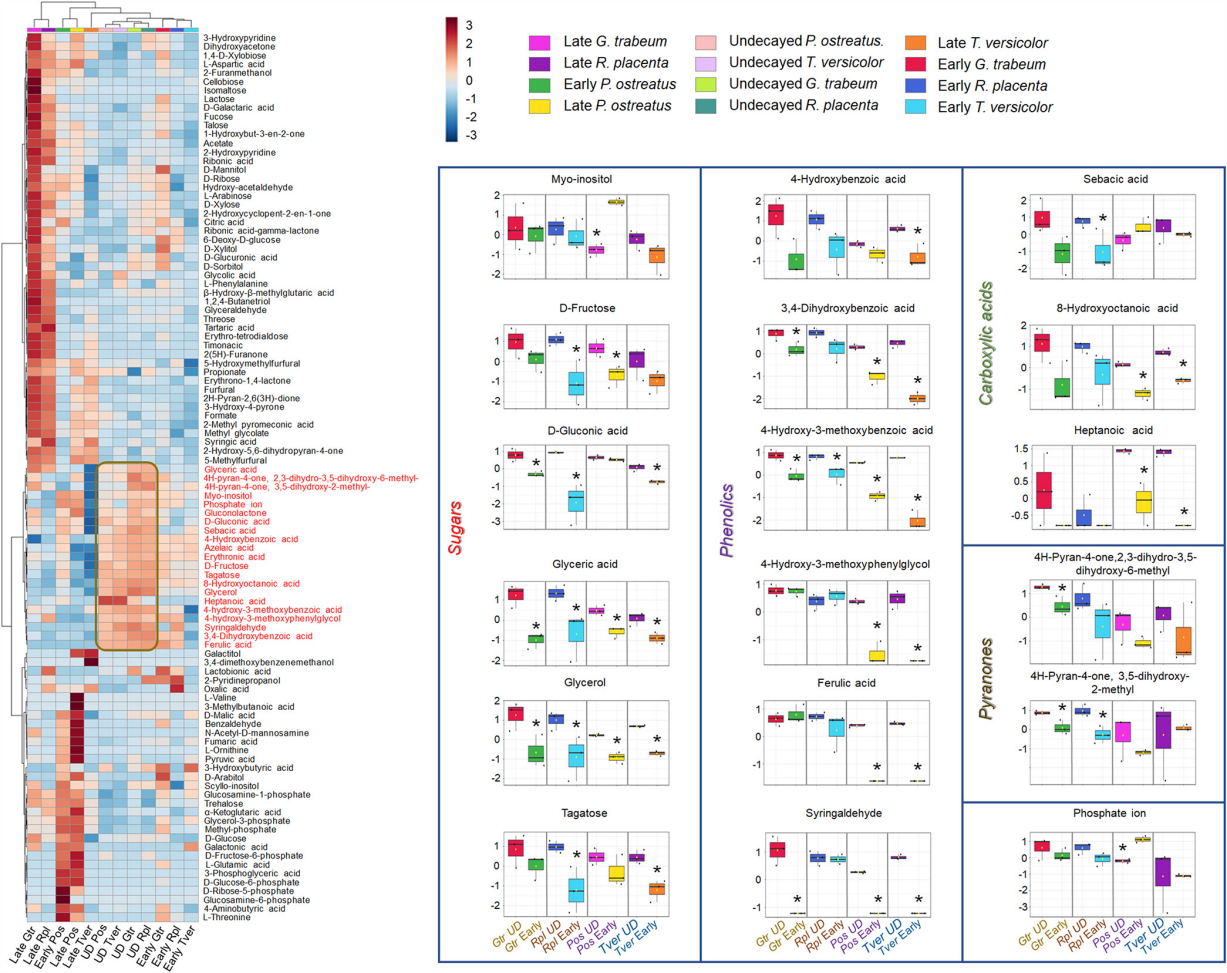
Heatmap showing 108 metabolites and their distribution among wood-decaying fungi (*G. trabeum*, *R. placenta*, *P. ostreatus*, and *T. versicolor*) at different stages of decay (undecayed, early, and late). Raw metabolite intensities were used in this case. The brown box identifies a group of 21 compounds (in red) that were highly abundant mainly in undecayed sections. The box plots on the right show 18 out of the 21 compounds that were highly abundant at undecayed sections, and whose abundance significantly changed (FC > 2, FDR < 0.05) in early wood decay sections. Most but not all the compounds were sugars and phenolics.

Another type of analysis in this study consisted of comparing compounds that were highly abundant in undecayed aspen wood and that were significantly reduced over the course of decay, particularly at early stages. Fig. 4 shows a heatmap (left) made with raw metabolite intensities in which it is evident that a small group of metabolites (21 compounds) was found at particularly high relative abundance in undecayed wood. Within this group there were several types of compounds such as sugars, phenolics, carboxylic acids, and pyranones whose content across decay sections varied depending on the type of fungus causing the decay. There were also compounds that were consistently degraded by all four fungi such as glyceric acid, glycerol, and 4-hydroxy-3-methoxybenzoic acid. These compounds are most likely diminished because of early sugar consumption at incipient wood decay, and phenolic compound detoxification. Remarkably, white rot fungi were considerably more efficient than brown rot fungi at removing phenolic compounds initially detected in undecayed wood; between the brown rot fungi, on the other hand, *R. placenta* was the least effective. Outstanding differences between brown and white rot fungi were particularly evident with ferulic acid and 4-hydroxy-3-methoxyphenylglycol that were barely degraded by brown rot fungi while being notably decreased by white rot. Most likely, this difference comes from the high expression of laccases at early decay stages in white rot fungi (13), and this initial “conditioning” or detoxification of the wood substrate probably helps boost white rot fungal growth in the early stages, perhaps giving them a competitive advantage during colonization by increasing inoculum potential. Some carboxylic acids such as 8-hydroxyoctanoic acid and heptanoic acid were also significantly decreased only in white rot fungi. Finally, contrary to what was observed for carboxylic acids and phenolics, pyranone compounds were significantly diminished only in brown rot fungi at early decay.

### Quenching of ROS at different wood decay stages as a key difference between brown and white rot fungi.

**FIG 5:**
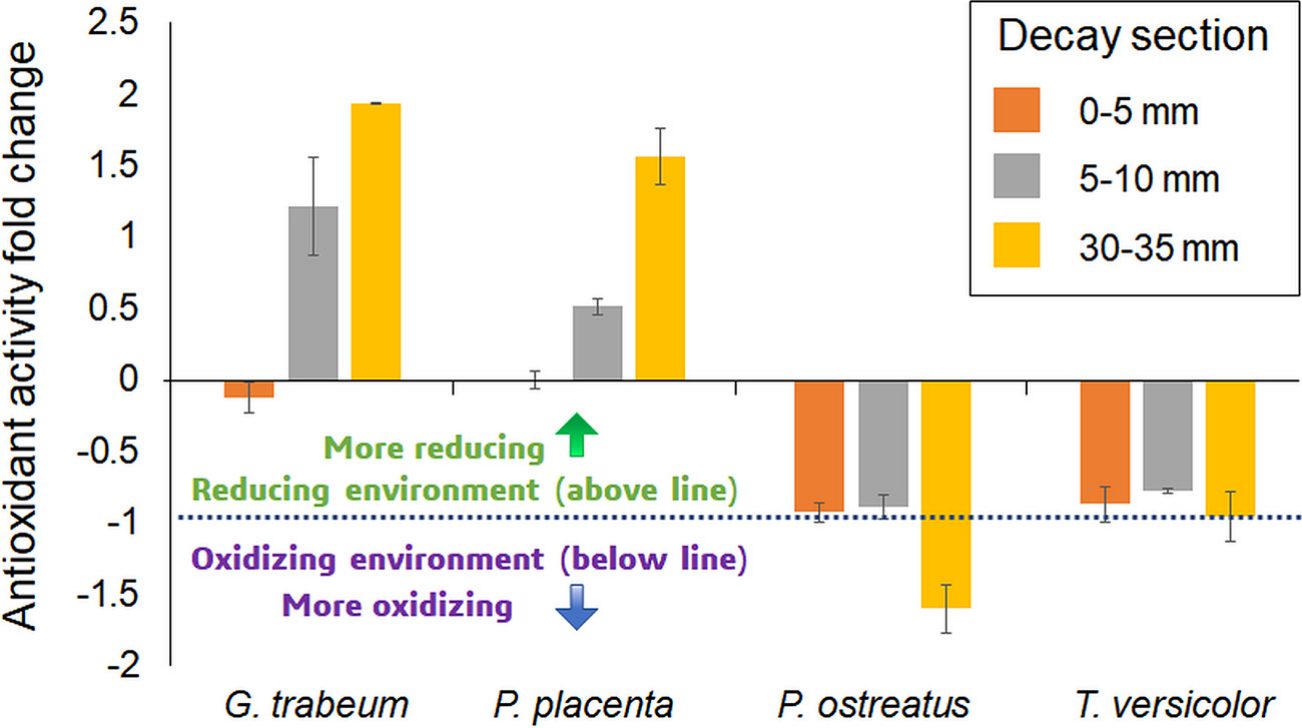
Antioxidant activity in the wood-degrading fungi used in this research across different wood decay stages. The dashed line indicates no antioxidant activity. The activity is expressed as a fold change with respect to the antioxidant activity found in undecayed wood, which was slightly reducing, as evidenced by the green background (reducing environment) at 0. Brown rot fungi showed antioxidant activity in all the decay stages with a sharp increase at the 5- to 10-mm and 30- to 35-mm sections. White rot fungi showed very low antioxidant activity, with a clear oxidative environment displayed at the 30- to 35-mm section for *P. ostreatus*.

Finally, we decided to determine the changes in antioxidant capacity during wood decay, which might also reflect some functional difference between different rot types and provide a deeper insight into the effects of different metabolome signatures. The evaluation of antioxidant activity (Fig. 5) showed that uncolonized wood has some small levels of antioxidant activity, which are then sharply increased in brown rot fungi and totally suppressed in white rot fungi, with *P. ostreatus* showing some oxidizing activity at late decay stages. This difference in antioxidant activity between brown and white rot fungi might be key to facilitate the use of highly oxidative radicals for lignocellulose decay while protecting fungal structures at different stages of decay. Furthermore, this strategy potentially contributes to accelerating decay in brown rot since the lignin structure could be loosened up by highly reactive hydroxyl radicals while enzyme damage is minimized. Hydrolytic enzymes can be particularly affected by ROS, and oxidation can adversely modify substrate-enzyme interactions (52). Therefore, this mechanism complements the previously shown increased of tolerance of ROS in some glycosyl hydrolases in brown rot compared to other fungal wood degraders (15, 52, 53), uncovering another crucial feature that distinguishes brown from white rot decay.

### Conclusion.

To our knowledge, this is the first *in planta* extracellular metabolomics study of wood-degrading fungi comparing white and brown rot organisms. The metabolite patterns showed a strong dependency with the type of wood rot and the stage of decay; late brown rot decay produced the most identifiable signature. For the white rot fungi evaluated, we could not identify a shared metabolite profile. Nonetheless, some commonalities between *P. ostreatus* and *T. versicolor* showed that galactitol could be a potential biomarker for white rot fungi. Additionally, we also identified that white rot fungi were more efficient at removing phenolic compounds that were originally present in undecayed wood. Globally, white rot fungi showed a higher capacity to release sugars early. However, this capacity did not increase at later decay stages. On the contrary, brown rot fungi were not able to release a lot of sugars from the wood substrate early during decay but at later stages showed a very sharp increase in the amount of released sugars. This probably reflects different life history strategies tailored for different environments where competition for resources is expected to be different. In addition, brown rot fungi reduce the risk of unwanted competition and “sugar stealing” with a higher production of secondary metabolites such as pyranones and furanones that could give them some competitive advantages and offset problems derived from a slower growth and enhanced release of nutrients.

## MATERIALS AND METHODS

### Fungal cultures.

Two brown rot fungi, *Rhodonia placenta* MAD 698-R (ATCC 44394) and *Gloeophyllum trabeum* (ATCC 11539), and two white rot fungi, *Trametes versicolor* A1-ATF (Forest Pathology culture collection, University of Minnesota, USA) and Pleurotus ostreatus (ATCC 32237), were used in this study. The fungi were cultured and maintained on potato dextrose agar (PDA) for routine experiments. For the wood wafer decay experiments, we followed the experimental set-up described by Zhang et al. (45). An overview of the process is shown in Fig. S1 in the supplemental material. Briefly, the fungi were grown on PDA plates for ~1 to 2 weeks, and small discs (5 mm) from the hyphal active growing edge were used to inoculate horizontally arranged wood strips (tongue depressors) in a soil jar. The soil mixture in the soil jar was composed of fertilizer-free topsoil, peat moss, and vermiculite (1:1:1) (obtained from local gardening stores) and hydrated with ~70% water (wt/vol). After an initial colonization period (2 to 3 weeks) at 25°C and 70% relative humidity, wet aspen wood wafers were propped up vertically on top of the developed “lawn” of hyphae. The soil jars were incubated in the dark for 2 to 4 weeks at 25°C and 70% relative humidity until the fungi had progressed ~70% of the distance up the wafers. Using the hyphal front to standardize the spatial location of initial decay, wood wafers were cut at 0 to 5 mm and 5 to 10 mm behind the front to represent early decay. For late decay, wafers were cut at 30 to 35 mm from the hyphal front. Nondecayed sections were cut 10 to 15 mm beyond (in front of) the hyphal front. A total of 36 wafers were colonized by each fungus, which constituted three pooled replicates for subsequent experiments (*n* = 12 wafers per pool).

10.1128/msphere.00545-22.2 FIG S1 Wafer culture setup used to separate undecayed, early, and late decay sections. Download FIG S1, TIF file, 1.0 MB. Copyright © 2022 Castaño et al. 2022 Castaño et al. https://creativecommons.org/licenses/by/4.0/ This content is distributed under the terms of the Creative Commons Attribution 4.0 International license.

### Metabolite extraction.

Each of the three pools of sections was extracted with a water:methanol (80:20) mixture after finely chopping, weighing, and adding wood sections to 15-mL Falcon tubes. Extraction solvent was added at a 1:3 ratio (sample weight [g]: solvent [mL]), and the mixture was subjected to vacuum for 5 min to help the solvent penetrate the wood pieces. After 60 min of agitation at 180 rpm and at 4°C, the samples were filtered and the flowthrough was stored at −20°C for subsequent experiments. The extracts are expected to be exclusively extracellular.as preliminary runs with fungal biomass grown in malt extract broth (data not shown) indicated this method did not produce significant extraction of intracellular metabolites.

### Metabolomics analysis.

For global metabolite analysis of polar compounds, 25 μL of the metabolite extracts were completely dried under vacuum. Dried extracts were chemically derivatized, as previously reported (54). Briefly, the extracted metabolites were derivatized by methoxyamination and trimethylsilyation (TMS), then the samples were analyzed by GC-MS on an Agilent GC 7890A using an HP-5MS column (30 m × 0.25 mm × 0.25 μm; Agilent Technologies, Santa Clara, CA) coupled with a single quadrupole MSD 5975C (Agilent Technologies). One microliter of sample was injected into a splitless port at constant temperature of 250°C. The GC temperature gradient started at 60°C, with a hold of temperature for 1 min after injection, followed by an increase to 325°C at a rate of 10°C/minute and a 5-min hold at this temperature. A fatty acid methyl ester standard mix (C8-28) (Sigma-Aldrich) was analyzed in parallel as standard for retention time calibration. GC-MS raw data files were processed using the Metabolite Detector software (55). Retention indices (RI) of detected metabolites were calculated based on the analysis of a fatty acid methyl ester (FAME) mixture, followed by their chromatographic alignment across all analyses after deconvolution. Metabolites were initially identified by matching experimental spectra to a Pacific Northwest National Laboratory (PNNL)-augmented version of Agilent GC-MS metabolomics library, containing spectra and validated retention indices for over 1,000 metabolites. Then, the unknown peaks were additionally matched with the NIST20/Wiley 11th edition GC-MS databases. All metabolite identifications and quantification ions were validated and confirmed to reduce deconvolution errors during automated data processing and to eliminate false identifications.

For analysis of volatile metabolites, extracts were mixed with methanol in a 1:1 ratio. A total of 120 μL of this mix were transferred to glass vials equipped with glass inserts for direct GC-MS analysis without chemical derivatization. One microliter of sample was injected into a splitless port at constant temperature of 240°C in the Agilent GC-MS system, as described above. A DB-WAX UI column (30 m × 0.25 mm × 0.25 μm; Agilent Technologies, Santa Clara, CA) was used for the analysis of volatile molecules. The GC temperature gradient started at 50°C, with a hold of temperature for 2 min after injection, followed by increase to 240°C at a rate of 20°C/minute and a 3.5-min hold. Processing was done using the Metabolite Detector software and identification and quantification ions were validated as explained above to eliminate false identifications and errors that can occur during automated data processing. Peak areas for identified metabolites in both methods of analysis (subject to chemical derivatization and directly injected in the case of volatiles) were obtained and further statistically analyzed.

The intensities obtained from the metabolomics analysis were normalized using ergosterol content in each decayed section. Ergosterol functions similarly to cholesterol in organismal plasma membranes, and its consistent concentration in the membranes of higher fungi makes it a standard biomarker for estimating fungal biomass. Ergosterol was quantified as previously described, using HPLC (56, 57). Because fungi have unique concentrations of ergosterol, per biomass, we further corrected the values obtained in the previous step using an ergosterol/dry biomass coefficient determined with biomass collected from liquid cultures using malt extract broth. The biomass from the liquid cultures was harvested according to the fungal growth rate observed on aspen wafers, cultured in soil jars as described before. The growth rate was recorded as mm of mycelia/day measured along the wafers. Finally, multivariate data analyses were carried out using MetaboAnalyst 5.0 (https://www.metaboanalyst.ca/MetaboAnalyst/).

### Overlay of antioxidant activity.

The antioxidant activity of the fungal samples was measured using the same wafer sections to enable overlay of early and late decay stage data. Specifically, antioxidant activity was measured using 2,2′-Azino-bis(3-ethylbenzothiazoline-6-sulfonic acid) (ABTS) according to Koleva et al. (58). Succinctly, a 7-mM ABTS solution was reacted for 16 h with 7 mM K_2_S_2_O_8_ to generate the cation radical ${\text{ABTS}}^{\text{•+}}$, which has a strong bright green color and absorbs light at 734 nm. Absorbance was adjusted with distilled water after the reaction to obtain a value close to 0.7. To measure antioxidant activity, 950 μL of ${\text{ABTS}}^{\text{•+}}$ were mixed with 50 μL of fungal extracts, and the absorbance at 734 nm was read after 10 min. The antioxidant activity was taken as the percentage of reduction of ${\text{ABTS}}^{\text{•+}}$ using the equation below:

$$\text{\% }\mathrm{antioxidant}\;\mathrm{activity}=\frac{\mathrm{Ab}s_{\mathrm{initial}}-\mathrm{Ab}s_{\mathrm{sample}}}{\mathrm{Ab}s_{\mathrm{initial}}}$$

where the $\mathrm{Ab}s_{\mathrm{initial}}$ and $\mathrm{Ab}s_{\mathrm{sample}}$ indicate the absorbance of the starting oxidized solution and the absorbance at the end of the experiment, respectively.

### X-ray scattering measurements.

The X-ray scattering measurements were performed at the Life Science X-ray Scattering (LiX) beamline of the National Synchrotron Light Source II, Brookhaven National Laboratory (Upton, NY). Data collection and processing were conducted as previously described (59). Scattering data from the wafers described in the text were recorded on two Pilatus pixel array detectors simultaneously, and later combined into intensity maps as functions of the scattering vector q and the azimuthal angle ϕ. The scattering intensity profiles were then extracted for comparison. The X-ray energy was 15.14 keV (0.819 Å wavelength). The beam size used for recording the data presented here was ~0.5 mm. This is significantly larger than the typical beam size (a few microns) used for tissue imaging experiments at LiX, and it was intentionally selected to average over possible structural variations within the wafers. Data were collected from two different locations on each sample to confirm reproducibility of the observed features.

## References

[1] Krah F-S, Bässler C, Heibl C, Soghigian J, Schaefer H, Hibbett DS. 2018. Evolutionary dynamics of host specialization in wood-decay fungi. BMC Evol Biol 18:119. doi:10.1186/s12862-018-1229-7. 30075699 PMC6091043

[2] Jutinico-Shubach LM, Castaño JD, Juarez T, Mariño M, Gómez-León J, Blandón LM. 2022. A novel basydiomycete isolated from mangrove swamps in the Colombian Caribbean shows promise in dye bioremediation. Bioremediat J 26:179–197. doi:10.1080/10889868.2021.1964426.

[3] Castaño JD, Crespo CC, Torres E. 2019. Evaluation of chemical and biological treatments to degrade oil palm empty fruit bunches (Elaeis guineensis Jacq.) and their potential use. J Oil Palm Res 31:271–280.

[4] Singh AP, Singh T. 2014. Biotechnological applications of wood-rotting fungi: a review. Biomass and Bioenergy 62:198–206. doi:10.1016/j.biombioe.2013.12.013.

[5] Suthar S, Kishore Singh N. 2022. Fungal pretreatment facilitates the rapid and valuable composting of waste cardboard. Bioresour Technol 344:126178. doi:10.1016/j.biortech.2021.126178. 34695588

[6] Cowling EB. 1961. Comparative biochemistry of the decay of sweetgum sapwood by white-rot and brown-rot fungi. USDA For Serv Tech Bull 1258:1–79.

[7] Filley TR, Cody GD, Goodell BS, Jellison J, Noser C, Ostrofsky A. 2002. Lignin demethylation and polysaccharide decomposition in spruce sapwood degraded by brown rot fungi. Org Geochem 33:111–124. doi:10.1016/S0146-6380(01)00144-9.

[8] Yelle DJ, Ralph J, Lu L, Hammel K. 2008. Evidence for cleavage of lignin by a brown rot basidiomycete. Environ Microbiol 10:1844–1849. doi:10.1111/j.1462-2920.2008.01605.x. 18363712

[9] ASTM International. 2007. Standard test method for wood preservatives by laboratory soil-block cultures. ASTM International. West Conshohocken, PA.

[10] Goodell B, Qian Y, Jellison J. 2008. Fungal decay of wood: soft rot-brown rot-white rot. p 9–31. *In* Schulzt TP, Militz H, Freeman MH, Goodell B, Nicholas DD (ed), Development of commercial wood preservatives, ACS symposium series. Washington, D.C.

[11] Wong DW. 2009. Structure and action mechanism of ligninolytic enzymes. Appl Biochem Biotechnol 157:174–209. doi:10.1007/s12010-008-8279-z. 18581264

[12] Floudas D, Binder M, Riley R, Barry K, Blanchette RA, Henrissat B, Martínez AT, Otillar R, Spatafora JW, Yadav JS, Aerts A, Benoit I, Boyd A, Carlson A, Copeland A, Coutinho PM, de Vries RP, Ferreira P, Findley K, Foster B, Gaskell J, Glotzer D, Górecki P, Heitman J, Hesse C, Hori C, Igarashi K, Jurgens JA, Kallen N, Kersten P, Kohler A, Kües U, Kumar TKA, Kuo A, LaButti K, Larrondo LF, Lindquist E, Ling A, Lombard V, Lucas S, Lundell T, Martin R, McLaughlin DJ, Morgenstern I, Morin E, Murat C, Nagy LG, Nolan M, Ohm RA, Patyshakuliyeva A, et al. 2012. The Paleozoic origin of enzymatic lignin decomposition reconstructed from 31 fungal genomes. Science 336:1715–1719. doi:10.1126/science.1221748. 22745431

[13] Zhang J, Silverstein KA, Castaño JD, Figueroa M, Schilling JS. 2019. Gene regulation shifts shed light on fungal adaption in plant biomass decomposers. mBio 10:e02176-19. doi:10.1128/mBio.02176-19. 31744914 PMC6867892

[14] Riley R, Salamov AA, Brown DW, Nagy LG, Floudas D, Held BW, Levasseur A, Lombard V, Morin E, Otillar R, Lindquist EA, Sun H, LaButti KM, Schmutz J, Jabbour D, Luo H, Baker SE, Pisabarro AG, Walton JD, Blanchette RA, Henrissat B, Martin F, Cullen D, Hibbett DS, Grigoriev IV. 2014. Extensive sampling of basidiomycete genomes demonstrates inadequacy of the white-rot/brown-rot paradigm for wood decay fungi. Proc Natl Acad Sci USA 111:9923–9928. doi:10.1073/pnas.1400592111. 24958869 PMC4103376

[15] Castaño JD, Zhang J, Anderson CE, Schilling JS. 2018. Oxidative damage control during decay of wood by brown rot fungus using oxygen radicals. Appl Environ Microbiol 84:e01937-18. doi:10.1128/AEM.01937-18. PMC6210117 30194102

[16] Miura D, Tanaka H, Wariishi H. 2004. Metabolomic differential display analysis of the white-rot basidiomycete Phanerochaete chrysosporium grown under air and 100% oxygen. FEMS Microbiol Lett 234:111–116. doi:10.1016/j.femsle.2004.03.017. 15109728

[17] Del Cerro C, Erickson E, Dong T, Wong AR, Eder EK, Purvine SO, Mitchell HD, Weitz KK, Markillie LM, Burnet MC, Hoyt DW, Chu RK, Cheng J-F, Ramirez KJ, Katahira R, Xiong W, Himmel ME, Subramanian V, Linger JG, Salvachúa D. 2021. Intracellular pathways for lignin catabolism in white-rot fungi. Proc Natl Acad Sci 118:e2017381118. doi:10.1073/pnas.2017381118. 33622792 PMC7936344

[18] Matsuzaki F, Shimizu M, Wariishi H. 2008. Proteomic and metabolomic analyses of the white-rot fungus Phanerochaete chrysosporium exposed to exogenous benzoic acid. J Proteome Res 7:2342–2350. doi:10.1021/pr700617s. 18435559

[19] Kijpornyongpan T, Schwartz A, Yaguchi A, Salvachúa D. 2022. Systems biology-guided understanding of white-rot fungi for biotechnological applications: a review. iScience 25:104640. doi:10.1016/j.isci.2022.104640. 35832889 PMC9272384

[20] Di Lella S, Tognetti R, La Porta N, Lombardi F, Nardin T, Larcher R. 2019. Characterization of silver fir wood decay classes using sugar metabolites detected with ion chromatography. J Wood Chem Technol 39:90–110. doi:10.1080/02773813.2018.1508301.

[21] Al-Bader N, Vanier G, Liu H, Gravelat FN, Urb M, Hoareau CM-Q, Campoli P, Chabot J, Filler SG, Sheppard DC. 2010. Role of trehalose biosynthesis in Aspergillus fumigatus development, stress response, and virulence. Infect Immun 78:3007–3018. doi:10.1128/IAI.00813-09. 20439478 PMC2897364

[22] Lustenhouwer N, Maynard DS, Bradford MA, Lindner DL, Oberle B, Zanne AE, Crowther TW. 2020. A trait-based understanding of wood decomposition by fungi. Proc Natl Acad Sci USA 117:11551–11558. doi:10.1073/pnas.1909166117. 32404424 PMC7261009

[23] Schilling JS, Kaffenberger JT, Held BW, Ortiz R, Blanchette RA. 2020. Using wood rot phenotypes to illuminate the “gray” among decomposer fungi. Front Microbiol 11:1288. doi:10.3389/fmicb.2020.01288. 32595628 PMC7303305

[24] Calonego FW, De Andrade MCN, Negrão D, Rocha CD, Minhoni Mt de A, Latorraca JV, Severo ETD. 2013. Behavior of the brown-rot fungus gloeophyllum trabeum on thermally-modified Eucalyptus grandis wood. Floresta e Ambient 20:417–423.

[25] Kobayashi H, Fukuoka A. 2013. Current catalytic processes for biomass conversion, p 29–52. *In* Suib SL. (ed), New and future developments in catalysis. Elsevier, Amsterdam, The Netherlands.

[26] Highley TL, Illman BL. 1991. Progress in understanding how brown-rot fungi degrade cellulose. Biodeterior Abstr 5:231–244.

[27] Plassard C, Fransson P. 2009. Regulation of low-molecular weight organic acid production in fungi. Fungal Biol Rev 23:30–39. doi:10.1016/j.fbr.2009.08.002.

[28] Kaddes A, Fauconnier M, Sassi K, Nasraoui B, Jijakli MH. 2019. Endophytic fungal volatile compounds as solution for sustainable agriculture. Molecules 24:1065. doi:10.3390/molecules24061065. 30889913 PMC6470890

[29] Kim MK, Nam P, Lee S, Lee K. 2014. Antioxidant activities of volatile and non-volatile fractions of selected traditionally brewed Korean rice wines. J Inst Brew 120. doi:10.1002/jib.180.

[30] Marynowski L, Goryl M, Bucha M, Smolarek-Lach J, Detman A, Sikora A, Chojnacka A, Simoneit BR. 2019. Trehalose, mannitol and arabitol as indicators of fungal metabolism in Late Cretaceous and Miocene deposits. Int J Coal Geol 201:51–61. doi:10.1016/j.coal.2018.11.003.

[31] 1995. Biosynthesis and biodegradation of hemicelluloses, p 33–70. *In* Singh A, Mishra P. (ed). Progress in industrial microbiology, vol 33. Elsevier, Amsterdam, The Netherlands.

[32] Ishizaki H, Hasumi K. 2014. Ethanol production from biomass, p 243–258. *In* Tojo S, Hirasawa T (ed), Research approaches to sustainable biomass systems. Academic Press, Cambridge, MA.

[33] Macabeo APG, Cruz AJC, Narmani A, Arzanlou M, Babai-Ahari A, Pilapil LAE, Garcia KYM, Huch V, Stadler M. 2020. Tetrasubstituted α-pyrone derivatives from the endophytic fungus, Neurospora udagawae. Phytochem Lett 35:147–151. doi:10.1016/j.phytol.2019.11.010.

[34] Ahluwalia V, Kumar J, Rana VS, Sati OP, Walia S. 2015. Comparative evaluation of two Trichoderma harzianum strains for major secondary metabolite production and antifungal activity. Nat Prod Res 29:914–920. doi:10.1080/14786419.2014.958739. 25248548

[35] Deutsch JC. 1998. Oxygen-accepting antioxidants which arise during ascorbate oxidation. Anal Biochem 265:238–245. doi:10.1006/abio.1998.2940. 9882398

[36] Henningson B. 1967. Physiology of fungi attacking birch and aspen pulpwood. Stud For Suec 52.

[37] Highley TL, Kirk TK. 1979. Mechanisms of Wood Decay and the Unique Features of Heartrots. Phytopathology 69:1151–1157. doi:10.1094/Phyto-69-1151.

[38] Goodell B, Qian Y, Jellison J, Richard M, Qi W. 2002. Lignocellulose oxidation by low molecular weight metal-binding compounds isolated from wood degrading fungi: a comparison of brown rot and white rot systems and the potential application of chelator-mediated fenton reactions, p 37–47. *In* Viikari L, Lantto R. (ed), Progress in biotechnology, Elsevier, Amsterdam, The Netherlands.

[39] Cho H-W, Kim SB, Jeong MK, Park Y, Miller NG, Ziegler TR, Jones DP. 2008. Discovery of metabolite features for the modelling and analysis of high-resolution NMR spectra. Int J Data Min Bioinform 2:176–192. doi:10.1504/ijdmb.2008.019097. 18767354 PMC3883573

[40] Shahbazi A, Zhang B. 2010. 5 - Dilute and concentrated acid hydrolysis of lignocellulosic biomass, p 143–158. *In* Waldron K.B., T.-B P. (ed). Woodhead publishing series in energy, Woodhead Publishing, Sawston, England.

[41] Mali T, Mäki M, Hellén H, Heinonsalo J, Bäck J, Lundell T. 2019. Decomposition of spruce wood and release of volatile organic compounds depend on decay type, fungal interactions and enzyme production patterns. FEMS Microbiol Ecol 95. doi:10.1093/femsec/fiz135. PMC6736282 31494677

[42] Zanellati A, Spina F, Bonaterra M, Dinuccio E, Varese GC, Scarpeci TE. 2021. Screening and evaluation of phenols and furans degrading fungi for the biological pretreatment of lignocellulosic biomass. Int Biodeterior Biodegradation 161:105246. doi:10.1016/j.ibiod.2021.105246.

[43] Presley GN, Zhang J, Schilling JS. 2018. A genomics-informed study of oxalate and cellulase regulation by brown rot wood-degrading fungi. Fungal Genet Biol 112:64–70. doi:10.1016/j.fgb.2016.08.004. 27543342

[44] Varela E, Tien M. 2003. Effect of pH and oxalate on hydroquinone-derived hydroxyl radical formation during brown rot wood degradation. Appl Environ Microbiol 69:6025–6031. doi:10.1128/AEM.69.10.6025-6031.2003. 14532058 PMC201180

[45] Zhang J, Presley GN, Hammel KE, Ryu J-S, Menke JR, Figueroa M, Hu D, Orr G, Schilling JS. 2016. Localizing gene regulation reveals a staggered wood decay mechanism for the brown rot fungus *Postia placenta*. Proc Natl Acad Sci USA 113:10968–10973. doi:10.1073/pnas.1608454113. 27621450 PMC5047196

[46] Mäkelä MR, Sietiö O-M, de Vries RP, Timonen S, Hildén K. 2014. Oxalate-metabolising genes of the white-rot fungus dichomitus squalens are differentially induced on wood and at high proton concentration. PLoS One 9:e87959. doi:10.1371/journal.pone.0087959. 24505339 PMC3914892

[47] Pizzolitto RP, Barberis CL, Dambolena JS, Herrera JM, Zunino MP, Magnoli CE, Rubinstein HR, Zygadlo JA, Dalcero AM. 2015. Inhibitory effect of natural phenolic compounds on *Aspergillus parasiticus* growth. J Chem 2015:1–7. doi:10.1155/2015/547925.

[48] Credille CV, Chen Y, Cohen SM. 2016. Fragment-based identification of influenza endonuclease inhibitors. J Med Chem 59:6444–6454. doi:10.1021/acs.jmedchem.6b00628. 27291165 PMC4948595

[49] Ramos-Ruiz R, Poirot E, Flores-Mosquera M. 2018. GABA, a non-protein amino acid ubiquitous in food matrices. Cogent Food Agric 4.

[50] Valli K, Wariishi H, Gold MH. 1990. Oxidation of monomethoxylated aromatic compounds by lignin peroxidase: role of veratryl alcohol in lignin biodegradation. Biochemistry 29:8535–8539. doi:10.1021/bi00489a005. 2271536

[51] Harvey PJ, Schoemaker HE, Palmer JM. 1986. Veratryl alcohol as a mediator and the role of radical cations in lignin biodegradation by Phanerochaete chrysosporium. FEBS Lett 195:242–246. doi:10.1016/0014-5793(86)80168-5.

[52] Castaño JD, Zhou M, Schilling J. 2021. Towards an understanding of oxidative damage in an α-L-Arabinofuranosidase of trichoderma reesei: a molecular dynamics approach. Appl Biochem Biotechnol 193:3287–3300. doi:10.1007/s12010-021-03594-w. 34125378

[53] Castaño J, Zhang J, Zhou M, Tsai CF, Lee JY, Nicora C, Schilling J. 2021. A fungal secretome adapted for stress enabled a radical wood decay mechanism. mBio 12. doi:10.1128/mBio.02040-21. PMC8406313 34399614

[54] Kim Y-M, Nowack S, Olsen MT, Becraft ED, Wood JM, Thiel V, Klapper I, Kühl M, Fredrickson JK, Bryant DA, Ward DM, Metz TO. 2015. Diel metabolomics analysis of a hot spring chlorophototrophic microbial mat leads to new hypotheses of community member metabolisms. Front Microbiol 6:209. doi:10.3389/fmicb.2015.00209. 25941514 PMC4400912

[55] Hiller K, Hangebrauk J, Jäger C, Spura J, Schreiber K, Schomburg D. 2009. MetaboliteDetector: comprehensive analysis tool for targeted and nontargeted gc/ms based metabolome analysis. Anal Chem 81:3429–3439. doi:10.1021/ac802689c. 19358599

[56] Newell SY, Arsuffi TL, Fallon RD. 1988. Fundamental procedures for determining ergosterol content of decaying plant-material by liquid chromatography. Appl Environ Microbiol 54:1876–1879. doi:10.1128/aem.54.7.1876-1879.1988. 16347700 PMC202764

[57] Presley GN, Schilling JS. 2017. Distinct growth and secretome strategies for two taxonomically divergent brown rot fungi. Appl Environ Microbiol 83:2987–2996. doi:10.1128/AEM.02987-16. PMC5359483 28130302

[58] Koleva II, Niederländer HAG, Van Beek TA. 2001. Application of ABTS radical cation for selective on-line detection of radical scavengers in HPLC eluates. Anal Chem 73:3373–3381. doi:10.1021/ac0013610. 11476238

[59] Yang L, Liu J, Chodankar S, Antonelli S, DiFabio J. 2022. Scanning structural mapping at the Life Science X-ray Scattering Beamline. J Synchrotron Radiat 29:540–548. doi:10.1107/S1600577521013266. 35254319 PMC8900859

