# Supplementary material for: Metabolomics Highlights Different Life History Strategies of White and Brown Rot Wood-Degrading Fungi
Source: mSphere. 2022 Dec 5;7(6):e00545-22. doi: 10.1128/msphere.00545-22 (PMC9769625; doi:10.1128/msphere.00545-22)
Supplement: TABLE S1 [file msphere.00545-22-s0003.docx]

**Table S1** Growth rates for different wood decaying fungi growing on an aspen wood wafers.

| **Fungus** | **Growth rate in aspen wood (mm/day)** |
| --- | --- |
| *G. trabeum* | 2.24 ± 0.24 |
| *R. placenta* | 2.50 ± 0.26 |
| *P. ostreatus* | 4.49 ± 0.46 |
| *T. versicolor* | 4.25 ± 0.32 |
