# Supplementary figures and images for: Metabolomics Highlights Different Life History Strategies of White and Brown Rot Wood-Degrading Fungi
Source: mSphere. 2022 Dec 5;7(6):e00545-22. doi: 10.1128/msphere.00545-22 (PMC9769625; doi:10.1128/msphere.00545-22)

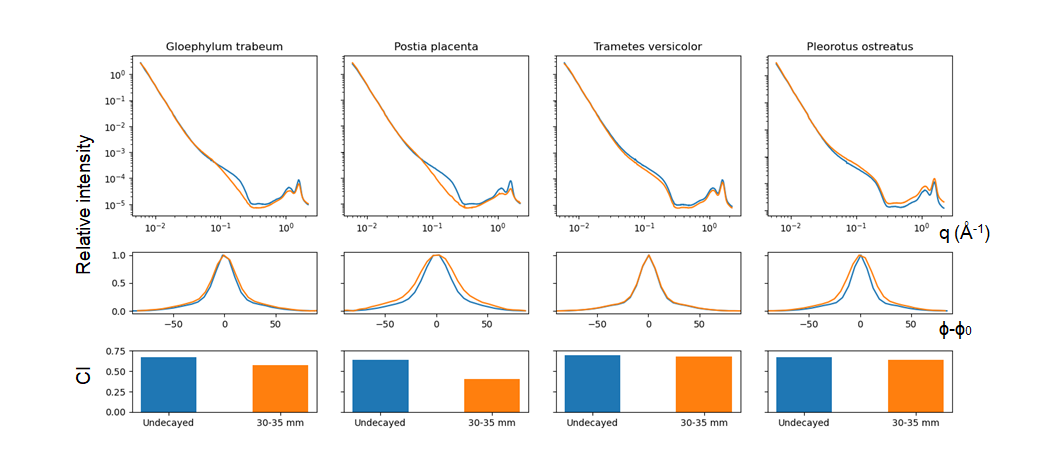

Supplement: FIG S2 [file msphere.00545-22-s0002.tif]

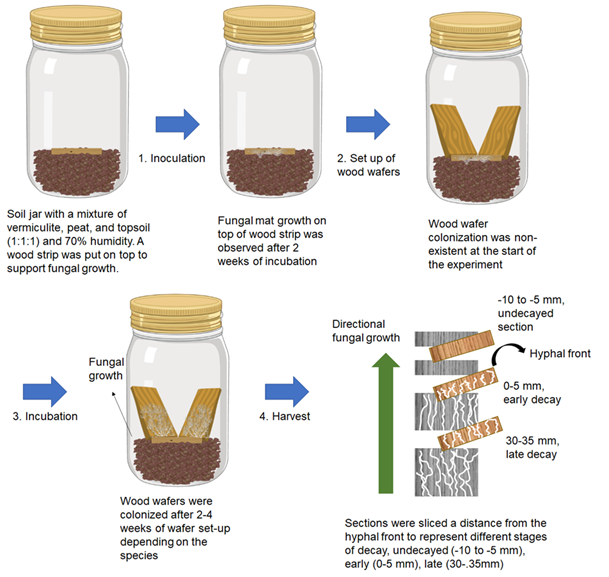

Supplement: FIG S1 [file msphere.00545-22-s0001.tif]
