# Supplementary material for: Metabolomics Highlights Different Life History Strategies of White and Brown Rot Wood-Degrading Fungi
Source: mSphere. 2022 Dec 5;7(6):e00545-22. doi: 10.1128/msphere.00545-22 (PMC9769625; doi:10.1128/msphere.00545-22)
Supplement: TABLE S2 [file msphere.00545-22-s0004.docx]

**Table S2** Compounds that were significantly (p<0.05) more abundant (FC ≥2) in either early or late decay stages in brown rot decay (G. trabeum and R. placenta) and white rot decay (P. ostreatus and T. versicolor).

| **Decay type** | **Early** | **Late** |
| --- | --- | --- |
| Brown rot decay |  | - 2H-Pyran-2,6(3H)-dione - Threose - Glyceraldehyde - Formate - Acetate - 3-Hydroxy-4-pyrone - D-Xylose - Lactose - L-Phenylalanine - Erythro-tetrodialdose - 2(5H)-Furanone - D-Xylitol - Ribonic acid-gamma-lactone - 1,2,4-Butanetriol - Timonacic - Ribonic acid - Fucose - Glyceric acid - L-Arabinose - β-Hydroxy-β-methylglutaric acid - Tartaric acid - 6-Deoxy-D-glucose - Glycolic acid |
| White rot decay | - Glycerol 3-phosphate - D-Malic acid - Fumaric acid - Trehalose - Glucosamine-1-phosphate - Scyllo-inositol - 2-Furanmethanol - Galactonic acid | - Glycerol 3-phosphate - Methyl phosphate - Scyllo-inositol - α-Ketoglutaric acid - Galactitol - 4-Hydroxypyridine - D-Arabitol |
