# Supplementary material for: Metabolomics Highlights Different Life History Strategies of White and Brown Rot Wood-Degrading Fungi
Source: mSphere. 2022 Dec 5;7(6):e00545-22. doi: 10.1128/msphere.00545-22 (PMC9769625; doi:10.1128/msphere.00545-22)
Supplement: TABLE S3 [file msphere.00545-22-s0005.docx]

**Table S3** Compounds that were significantly (p<0.05) more abundant (FC ≥2) in either early or late decay stages in G. trabeum, R. placenta, P. ostreatus, and T. versicolor.

| **Fungus** | **Early** | **Late** |
| --- | --- | --- |
| *G. trabeum* | - D-Arabitol - Lactobionic acid | - Furfural - 2H-Pyran-2,6(3H)-dione - Glycolic acid - 5-Hydroxymethylfurfural - 3-Hydroxy-4-pyrone - Erythrono-1,4-lactone - Timonacic - Ribonic acid - 2-Methyl pyromeconic acid - 2-Hydroxy-5,6-dihydropyran-4-one - Erythro-tetrodialdose - Glyceric acid - Talose - Tartaric acid - β-hydroxy-β-methylglutaric acid - Glyceraldehyde - Methyl glycolate - Isomaltose - 1,2,4-butanetriol - Acetate - Ribonic acid-gamma-lactone - 2(5H)-Furanone - 5-Methyl furfural - Cellobiose - 1,4-D-Xylobiose |
| *R. placenta* | - Oxalic acid | - Propionate - Methyl glycolate - Hydroxy-acetaldehyde - D-Glucuronic acid - Timonacic - Tartaric acid - 1-Hydroxybut-3-en-2-one - 1,2,4-Butanetriol - Acetate - 1,4-D-xylobiose - Erythrono-1,4-lactone - 2-Hydroxypyridine - 5-Methyl furfural - 3-Hydroxy-4-pyrone - Ribonic acid - Threose - Erythro-tetrodialdose - Formate - 2-Hydroxy-5,6-dihydropyran-4-one - L-Aspartic acid - Syringic acid - 2-Methyl pyromeconic acid - β-Hydroxy-β-methylglutaric acid - 2H-Pyran-2,6(3H)-dione |
| *P. ostreatus* | - 6-Deoxy-D-glucose - Glycolic acid - L-Arabinose - Scyllo-inositol - D-Glucuronic acid - Glucosamine-6-phosphate - 4-Aminobutyric acid (GABA) - D-Ribose - Glucosamine-1-phosphate - Myo-inositol - 3-Phosphoglyceric acid | - 5-Methylfurfural - N-Acetyl-D-mannosamine - 3-Methylbutanoic acid - L-Valine - Formate - L-Ornithine - Galactitol - Syringic acid |
| *T. versicolor* | - Glycerol 3-phosphate - Galactonic acid - D-Malic acid - 4-Hydroxypyridine - 3-Hydroxybutyric acid - 2-Furanmethanol - Fumaric acid - Glucosamine-1-phosphate | - Syringic acid - Formate - Threose - 2-Methyl pyromeconic acid - Oxalic acid - 3,4-Dimethoxybenzenemethanol (Veratryl alcohol) - Timonacic - Galactitol - Erythrono-1,4-lactone |
